# Ethical, Legal, and Practical Concerns Surrounding the Implemention of New Forms of Consent for Health Data Research: Qualitative Interview Study

**DOI:** 10.2196/52180

**Published:** 2024-08-07

**Authors:** Svenja Wiertz, Joachim Boldt

**Affiliations:** 1 Department of Medical Ethics and the History of Medicine University of Freiburg Freiburg Germany

**Keywords:** health data, health research, informed consent, broad consent, tiered consent, consent management, digital infrastructure, data safety, GDPR

## Abstract

**Background:**

In Europe, within the scope of the General Data Protection Regulation, more and more digital infrastructures are created to allow for large-scale access to patients’ health data and their use for research. When the research is performed on the basis of patient consent, traditional study-specific consent appears too cumbersome for many researchers. Alternative models of consent are currently being discussed and introduced in different contexts.

**Objective:**

This study explores stakeholder perspectives on ethical, legal, and practical concerns regarding models of consent for health data research at German university medical centers.

**Methods:**

Semistructured focus group interviews were conducted with medical researchers at German university medical centers, health IT specialists, data protection officers, and patient representatives. The interviews were analyzed using a software-supported structuring qualitative content analysis.

**Results:**

Stakeholders regarded broad consent to be only marginally less laborious to implement and manage than tiered consent. Patient representatives favored specific consent, with tiered consent as a possible alternative. All stakeholders lamented that information material was difficult to understand. Oral information and videos were mentioned as a means of improvement. Patient representatives doubted that researchers had a sufficient degree of data security expertise to act as sole information providers. They were afraid of undue pressure if obtaining health data research consent were part of medical appointments. IT specialists and other stakeholders regarded the withdrawal of consent to be a major challenge and called for digital consent management solutions. On the one hand, the transfer of health data to non-European countries and for-profit organizations is seen as a necessity for research. On the other hand, there are data security concerns with regard to these actors. Research without consent is legally possible under certain conditions but deemed problematic by all stakeholder groups, albeit for differing reasons and to different degrees.

**Conclusions:**

More efforts should be made to determine which options of choice should be included in health data research consent. Digital tools could improve patient information and facilitate consent management. A unified and strict regulation for research without consent is required at the national and European Union level. Obtaining consent for health data research should be independent of medical appointments, and additional personnel should be trained in data security to provide information on health data research.

## Introduction

### Background

In the European Union (EU), the outlines of a European health data space are currently being developed and discussed. It aims to facilitate accessing health data for research purposes, among other features [1]. In some countries, such as Estonia and Finland, fully digitalized health data infrastructures that also allow for secondary use of health data for research have already been implemented. In other countries, among them Germany, developments are ongoing [2-7]. In Germany, a central example of such developments is the government’s medical informatics initiative (MII; *Medizininformatik-Initiative*) [8]. Its purpose is to create data integration centers at German university medical centers and to establish centralized access to the data.

In regard to these infrastructures, it is an important question from ethical, legal, and social perspectives whether and how patients are informed about the use of their data and how they consent to this use [9-11]. Informed consent consists of 2 elements, as the term indicates: information ensures that patients know what their health data are used for. From an ethical point of view, information can be regarded as a demand for the principle of transparency. By contrast, consent safeguards the ability to control what one’s data are used for and serves to retain privacy. Together, transparency and control allow autonomy rights to be exercised meaningfully.

Various models of informed consent could be used for health data research. *Specific consent*, the standard in research involving humans, demands that trial participants are informed about and consent to the specific aims and methods of the trial that they take part in. Applied to health data research, this would mean that patients are informed about and consent to each health data research project that accesses and uses the patient’s data. Since this would place major obstacles in the way of research, among other drawbacks (compare in the Previous Research section), a number of alternatives are discussed, such as broad consent, tiered consent, meta-consent, and dynamic consent.

In *broad consent* patients consent to the use of their health data for research purposes in unspecified future research. The model of *tiered consent* enables patients to tailor individual consent profiles through the selection of broad and narrow options for data use (*tiers*) and thus allows them to adjust their preferences regarding the kinds of future research to which they are willing to consent. The *dynamic consent* framework facilitates study-specific consent by implementing consent requests and information on a web-based platform. Similarly, *meta-consent* implements tiered consent on a web-based platform, including options to ask for study-specific consent or to opt for broad consent [12-15].

The General Data Protection Regulation (GDPR), which was introduced in the EU in 2018, constitutes an important part of existing governance frameworks. Although it contributes to harmonizing legal approaches to data sharing in member states, it does not prescribe a specific model of consent when consent is used as a basis to legally justify the use of health data for research. In fact, under certain conditions, the GDPR permits accessing health data for research without consent [16,17]. Article 9, paragraph 2, of the GDPR allows EU member states to issue such exemptions for scientific research. The prerequisite is that the processing of personal health data is necessary for the purpose of the research and that safeguards are in place to protect the rights of the “data subjects” in accordance with Article 89, paragraph 1. In addition, the processing must be proportionate to the objective pursued. In Germany, section 27 of the German Federal Data Protection Act constitutes such a national regulation.

The World Medical Association’s Declaration of Taipei introduces consent as a necessary condition for using health data in research involving humans with few exemptions and lists information that must be included in informed consent for data sharing and biobanking [18]. These stipulations allow for but do not require specific consent for data stored in repositories that are designed for “multiple and indefinite uses.” They are compatible with other models of consent as well.

The German MII continues to consider consent as the appropriate legal basis for authorizing the secondary use of health data for research purposes, as is standard in many other EU countries [3,8]. More specifically, the initiative opts for a standardized model of broad consent. All participating medical centers are asked to use the same information and consent form, to which optional modules (eg, concerning biobanking) can be added according to local needs. These developments raise the question of how informed consent should be implemented in health data research infrastructure and which model of consent is to be favored. Moreover, the first experiences of stakeholders in this implementation process can provide valuable insight and information on the challenges connected to operationalizing informed consent in the context of health data research.

### Previous Research

Informed consent safeguards transparency and enables patients to exert control over the use of their personal health data and retain privacy. In the ethical debate on health data research, informed consent thus plays an important role. In addition, in the GDPR, obtaining consent is the standard way to legally justify the use of personal health data for research, although consent can be waived if member states fulfill certain conditions (compare in the Background section).

In the current scholarly ethical debate, there is wide agreement that specific informed consent, an ethical cornerstone of clinical research, has significant drawbacks when applied to biobank and health data research [12]. For a number of reasons, staying with specific consent in the case of health data research appears problematic. Since valuable data research will often involve data from a large number of patients, obtaining consent will be cumbersome and delay or prevent studies [12]. This may impede studies and diminish the positive impact that these studies could have had on health and society. Implementing specific consent on a digital platform may mitigate these problems, but it will still constitute an obstacle to large-scale data research. In addition, patients themselves might be irritated by being asked to consent to data research for single studies if approached repeatedly for a large number of studies [19,20]. As some surveys show, many patients and citizens generally deem the use of their health data for research acceptable. These surveys also show that while patients and citizens might prefer traditional specific consent, they deem other forms of consent acceptable [21-24]. Finally, although there are serious risks connected to the use of health data, such as loss of data and misuse, these risks are different and arguably less severe than the risks of clinical research [12].

Accordingly, in the conceptual ethical debate about alternative consent models for data- or biospecimen-based research, *broad consent* to unspecified future research is suggested as an alternative to specific consent [12,15]. The main issues highlighted as problematic in this debate so far regard the problem of consenting to research that is as of yet unknown. The information conveyed at the time of obtaining consent is necessarily restricted to general information on risks and opportunities of accessing and storing health data, pseudonymization, and anonymization procedures, data security principles, and features of typical health data research projects. By contrast, no information can be supplied concerning specific research projects, their goals and methods, and their risks. Since the validity of consent is based on the knowledge of what one consents to and since broad consent information is necessarily scarce, the validity of this consent is called into question [14,25,26]. Indeed, the provocative question of whether broad consent can be counted as informed consent at all has been raised [27].

Various other proposals have been made as to how the goal of relieving researchers and research participants of the need for frequent and time-consuming information and consent processes can be achieved while at the same time maintaining a high standard of informational self-determination [13]. Making use of digital infrastructure for collecting and managing consent has been a prominent suggestion in this debate. The *dynamic consent* framework has been discussed as a possible way forward that could allow for study-specific consent through a web-based platform, which would facilitate the process of obtaining consent. The suggested framework of meta-consent combines this idea with the principle of tiered consent, in which patients are enabled to tailor their individual consent profiles through the selection of broad and narrow options for data use (tiers), and allowed to adjust their preferences flexibly from home through a web-based platform [28-32] (for our interviews, we have separated the issues of consent models from their digital or analog implementation. We are thus not discussing meta-consent and dynamic consent by name but are referring to the same concepts when we discuss options of a digital implementation of specific or tiered consent).

With a focus on Europe, it has been discussed how GDPR-based rights can be safeguarded and implemented as part of biobank consent management procedures. These rights include the right to be informed about the collection and use of personal data, the right to rectification of wrong personal data, and the right to erasure of personal data [33]. Since research on specimens always also involves the use of health data, the GDPR is a relevant legal framework for this kind of research as well as for research solely on health data. The findings of this debate thus are generally relevant for health data research as well. Nevertheless, the GDPR biobank debate does not touch upon possible alternative forms of consent. In biobank research today, broad consent is the standard and widely accepted model of consent [15].

Attitudes toward data sharing for health research have been studied in the United States as well as in Europe, and some meta-studies have attempted to summarize the results [22,34-37]. They not only report widespread support for data sharing for research purposes under certain conditions (eg, data security, trust, control, and privacy) but also highlight ethnocultural differences in attitude [38]. Concerns identified relate to sharing of data (or specimens) with for-profit organizations [22] as well as transferring data across borders, especially from EU countries to non-EU countries [37].

For Germany in particular, acceptance of broad consent has been studied among outpatients of a university hospital in northern Germany [23] as well as among participants of a cancer registry in the state of Baden-Württemberg [24]. Both studies observe differing levels of acceptance of broad consent (58.9% vs 86.9%, respectively). In a qualitative study from Germany on the perception of risks and opportunities of health data research in general, stakeholder groups from health research, health care, medical informatics, patient advocacy groups, and politics were interviewed. Regarding informed consent, the study concludes that some stakeholders are afraid that patients may be unduly influenced to provide consent if they are, as patients, under distress or wrongly assume that health data research will directly benefit them [39]. The study does not differentiate between models of consent. Overall, although, on a conceptual level, the pros and cons of models of consent regarding health data research have been debated, there are no studies empirically examining the acceptance and perceptions of advantages, disadvantages, and implementation challenges of different models of consent from stakeholder perspectives.

### Objective

In our study, we build on and complement the aforementioned studies by focusing on different models of consent and consent procedures. Different models of consent for health data research have been developed on the basis of conceptual and normative arguments. So far, empirical studies and surveys have not taken up these models and compared their acceptance and perception of drawbacks and opportunities from the perspective of stakeholder groups. We aimed to explore the perception of drawbacks and opportunities of different models of consent among stakeholders from patient associations, data protection, medical informatics, and medical research.

The study is explorative. We assume that the perspectives of stakeholders who are dealing with health data research and informed consent in different roles and with different tasks can help to uncover blind spots in the debate and challenges in practical application that have not received adequate attention in the conceptual, ethical debate on consent models for health data research so far.

## Methods

### Overview

We interviewed stakeholders who are and will be affected by the implementation of informed consent regarding health data research and can be assumed to have a perspective on the drawbacks and opportunities of consent models. Medical researchers will use the relevant infrastructures and data and will have to abide by consent provisions. Patients are the ones whose individual rights and interests are meant to be protected by consent. Data protection officers safeguard that data handling procedures, including consent procedures, conform to the law. Health IT specialists implement and run the IT infrastructure for storing and managing health data and research requests, as well as consent specifications. We included 3 to 4 participants from each stakeholder group, 13 participants in total.

For the profession-based groups, we focused on stakeholders with ties to German university medical centers. These centers are currently implementing broad consent as devised by the MII. Their employees can thus be assumed to have a level of experiential and expert knowledge that enables them to take a stance on models of consent and their implementation.

Representatives of these groups were identified via a web-based search and contacted by publicly available email addresses. The selection criteria were to include participants who are familiar with the topic of informed consent (documented statements, publications, participation in relevant conferences or workshops, etc) and to ensure diversity of individuals and perspectives within the groups (geographical location, specific area of expertise, and gender). Our group of medical researchers comprised 2 physician researchers and 1 epidemiologist.

To recruit patient representatives familiar with the topics of consent and research, as well as patients’ attitudes beyond their own, we checked and contacted several established German patient associations and invited individuals, on the basis of either publicly available records of experience or recommendations by board members of the associations.

### Ethical Considerations

The study was approved by the ethics commission of the University of Freiburg (approval number 21-1701). It was conducted in accordance with applicable German and EU data protection regulations. Participation was voluntary and not compensated. All interviewees were informed about the aims, methods, benefits, and risks of participating in the study and gave documented informed consent. Any information allowing personal identification was removed from the interview transcripts before analysis. All data in the publication, as well as any data shared on request, were anonymized.

### Interviews

We conducted qualitative, semistructured interviews with the respective stakeholders. We opted for group interviews in order to profit from the effect of homogeneous focus groups to illuminate the topic in breadth and depth [40].

Of all the persons interviewed (N=13), the patient representatives (n=3, 23%), the medical researchers (n=3, 23%), and the health IT specialists (n=4, 31%) were interviewed in groups as planned. For the data protection officers (n=3, 23%), 3 individual interviews had to be conducted due to organizational reasons.

The interviews took place between May and December 2022 in a videoconference format. All interviews were jointly conducted by both authors. The interview guide consisted of a series of general questions and some group-specific questions. One interview session lasted between 60 and 150 minutes. The interviews were recorded and subsequently transcribed by an external service provider.

The interview guide was prefaced by a short explanation of broad consent, tiered consent, specific consent, and digital consent platforms (Multimedia Appendix 1). The guide included 8 questions that were addressed to all groups equally, plus 4 to 8 group-specific questions. The explanatory text and the questions were sent to the participants by email several days before the interview.

### Data Analysis

The transcriptions were analyzed in an iterative process using qualitative content analysis. We applied the structuring qualitative content analysis as developed by Mayring [41] and Kuckartz [42]. The software MAXQDA (version 22.61; VERBI Software GmbH, 2022) was used for coding. Given the small number of participants in each group, no quantitative analysis was carried out. No interrater reliability or percent level of agreement were calculated.

The coding categories of the material were initially systematized according to action steps in the process of devising and carrying out a data research project: from planning the research to obtaining consent, adapting and withdrawing consent, to handling of consent data and treatment data (in research contexts), to handling of research results, and to addressing data loss and misuse. The category scheme according to which the material was sorted was developed before the analysis and applied deductively. Where necessary, it was later adapted inductively based on the content of the material. The identification of topics, problem areas, and proposed solutions was carried out inductively using the sorted material.

All items were annotated with a 1-sentence summary. On the basis of these summaries, a table was created to grant an overview of the topics of the interview (Multimedia Appendices 2 and 3). For this purpose, suggestions for solutions as well as objections in regard to either the problem itself or a suggested solution were grouped with the problems they addressed. In some cases, solutions were offered to implied problems without the problems themselves being named. The implied problems were added to the table where needed. This sorting was conducted by SW, and JB reviewed all resulting categories. Any disagreements regarding either the placement of a particular statement in a category or the usefulness or exact borders of the categories were discussed between the 2 researchers until a consensus was reached.

## Results

### Overview

Our interviewees brought up a large number of problems, concerns, and possible solutions regarding models of consent and their implementation. A complete overview of the results can be found in Table S1 in Multimedia Appendix 2 and Table S1 in Multimedia Appendix 3.

The following representation of results highlights topics that either are addressed repeatedly by different stakeholder groups or have not received much attention in the existing ethical debate on models of consent and can thus be considered new and potentially worthy of further consideration.

### Attitudes on Specific and Broad Consent

When discussing models of consent for health data research, our interviewees first focused on broad consent and specific consent. The expressed points of view coincide in many respects with the current state of the scholarly debate on this topic. For example, patient representatives doubted that broad consent conveyed all relevant information and prima facie favored specific consent. Data protection officers agreed that specific consent was better suited than broad consent to inform patients adequately, while they deemed broad consent more advantageous for researchers. Researchers pointed to the amount of work needed to design and obtain specific consent and to obtain project-specific ethics approval. They claimed that in order to be workable, biobanks and large health data registries presupposed broad consent solutions for research:

Also natürlich, vielmehr auch spezifisch eigentlich spricht mich prinzipiell am meisten an, weil ich dann am ehesten die Klarheit habe, wofür genau, für welches Ziel und wer und in welchem Rahmen und so.

Patient representative 1

[So of course, it’s specific that appeals to me the most in principle, because that’s when I have the most clarity about what exactly, for what goal and who and in what context and so on.]

Patient representative 1, translation by SW

Umgekehrt haben wir natürlich bei den projektspezifischen Dingen allein so viel Reibungs- oder Zeitverluste, dass sie durch die Ethik durchmüssen, das dreimal überarbeiten müssen und darüber verliert man auch viel. Und deswegen ist die spezifische Aufklärung manchmal schon durchaus auch ein Problem...

Medical researcher 3

[Conversely, of course, with project-specific things alone we have so much friction and loss of time; they have to pass through ethics, they have to be revised 3 times and thereby there is a lot of loss as well. And therefore specific consent is indeed sometimes also a problem...]

Medical researcher 3, translation by SW

As a less obvious point, both data protection officers and IT specialists remarked that designing and implementing broad consent could be very complex and time-consuming as well. Data protection officers mentioned the standard broad consent form of the MII as an example, as it contains a number of modules that can but need not be included in local applications. They pointed out that MII broad consent came in regularly updated versions that needed to be tracked and offered some yes or no options for patients (MII yes or no options include transferring data to countries without EU adequacy decision, access to data from outpatient care, transferring data to and from other medical research facilities, and being contacted in case of incidental findings). They also referred to requests to data use and access committees, pseudonymization, and related data management issues that remain laborious when using broad consent. IT specialists stressed the costs of implementing broad consent and the labor needed to establish consent procedures that were not project specific. They also stressed that once established, the efficiency of digital consent implementation of either broad consent or other consent models could be high:

Gerade, wenn wir jetzt Medizininformatik noch mal berücksichtigen, die verschiedenen Einwilligungsversionen, also das macht es extrem komplex. Ich habe einen Patienten, der hat meinetwegen die 1.6D-Variante unterschrieben, hat aber nicht mehr die 1.72 unterschrieben. Das muss ich alles irgendwo ab- oder möchte man abbilden, muss man aus meiner Sicht auch abbilden.

Data protection officer 1

[In particular, if we look at the medical informatics initiative once again, the different versions, so that makes it extremely complex. I have a patient, who has, let’s assume, signed version 1.6D, but hasn’t signed 1.72 anymore. One has to—or one wants to, has to in my opinion, map that.]

Data protection officer 1, translation by SW

Aber ich meine, die meisten Lösungen, die ich kenne, die versuchen jetzt alles, also sowohl die klinischen als auch die studienspezifischen als auch den Broad Consent über so eine selbe Plattformlösung abzubilden, und dann hat man natürlich vereinheitlichte Prozesse. Dann kann man da sehr effektiv mit umgehen.

Health IT specialist 3

[But I think, most of the solutions I know, they attempt to do it all, so the clinical, as well as the study specific, as well as the broad consent—to map it all over the same platform solution. And then one has standardized processes. Then one can work very effectively.]

Health IT specialist 3, translation by SW

### Tiered Consent

Tiered consent is the least well-known model among our participants. Nevertheless, it was regarded by some as the best option to allow for something broader than specific consent while still providing patients with a good degree of control over their data:

ist aus meiner Sicht das gestufte Modell natürlich, also wenn man schon, sagen wir mal so, in die Forschung grundsätzlich einwilligt als Patient, dann ist das gestufte Modell natürlich das mit der besten Flexibilität, gegebenenfalls mit der höchsten Transparenz.

Patient representative 2

[From my point of view, of course, if one, let’s say, generally consents to research as a patient, then the tiered model is of course the one with the best flexibility, possibly with the highest transparency.]

Patient representative 2, translation by SW

It was identified as somewhat less favorable for data research assuming the resulting average permission for the use of data would be narrower than for broad consent. Concerns regarding the efforts needed to manage individual consent profiles were raised. Broad consent, as suggested by the MII in Germany, is seen as not far removed from a model of tiered consent, as it offers options for patients to express preferences (compare in the Attitudes on Specific and Broad Consent section).

### Comprehensibility

Lack of comprehensibility of information material was named by all groups as an important obstacle to obtaining adequate consent, regardless of which consent model is chosen. IT representatives and researchers generally ascribed a lack of comprehensibility to legal requirements, including GDPR requirements:

Also die Vorgaben...die...für Einwilligungserklärungen vorgegeben werden, die sind nicht mehr leicht verständlich. Die sind sehr komplex. Da sind sehr viele Rechtstexte drin. Also das braucht mir niemand erzählen, dass es Teilnehmer gibt, die sich das bis zum Ende wirklich exakt durchlesen und dann auch noch verstanden haben, das glaube ich nicht.

Health IT specialist 2

[As in, those guidelines...for consent forms, they are no longer easy to understand. They are highly complex. There is a lot of legal text in them. Like, no one has to tell me that there are participants who actually read this in detail, through to the end, and after that have really understood it. I don’t believe it.]

Health IT Specialist 2, translation by SW

One interviewed data protection officer pointed out that while data protection information may often, by habit, make use of complicated legal terminology, the GDPR explicitly asks for comprehensible information. Solutions offered include oral information by specifically trained personnel alone or together with researchers (for more details concerning this point, see the Expertise and Professional Background of Persons Obtaining Consent section).

Videos on specific data protection issues (such as pseudonymization and anonymization) were regarded by patient representatives and researchers as valuable supplements to in-person information, especially because these materials provide the opportunity for patients to become acquainted with relevant issues at home or in other places distinct from the clinical and therapeutic context.

Finally, IT specialists and data protection officers welcomed the option of web-based patient information sites on health data research as a tool to ameliorate information transfer. Data protection officers argued that such a platform could be used to recruit patients for health data research as well, which could lead to more active involvement of patients:

Und dann kann man das noch größer denken. Dann kann man eine solche Plattform, Transparenz, kann man so gestalten, dass die Bürger sich erkundigen können: Was gibt es für Forschungsprojekte? Dann können wiederum Forscher in dieser Plattform ihre Forschungsprojekte präsentieren. Und die Bürger können sich aktiv bewerben darum und sagen: Kann ich da nicht mitmachen bei dem Forschungsprojekt?

Data protection officer 2

[And then one can think that on a bigger scale. One could create such a platform, transparency, fashion it in a way that the citizens can inform themselves: what research projects are there? And then the researchers can use the platform to present their projects. And citizens can actively apply for them and say: Can I join in this research project?]

Data protection officer 2, translation by SW

### Expertise and Professional Background of Persons Obtaining Consent

Patient representatives were reluctant to accept researcher physicians as the sole contact persons for obtaining in-person health data research information. This is due to suspected partiality and lack of relevant data protection expertise. As a consequence, patient representatives in our interviews preferred researcher-independent information providers with specific data expertise in addition to researchers providing information:

Also ich stelle mir schon vor, dass die Aufklärung sehr gut ist und differenziert ist und verständlich ist, also dass mir ein Datenschutzmensch und ein Computermensch erklären, worin die Chancen und Risiken bestehen, dass mir ein medizinisch geschulter Mensch erklärt, was der Sinn dieser ganzen Sache ist und auch mir den Ablauf erklären kann…Aber für mich ist immer wichtig, dass es auch aus kritischer Seite beleuchtet wird. Wenn da nur Befürworter sozusagen Propaganda mir liefern [lacht], dann fühle ich mich nicht wirklich aufgeklärt.

Patient representative 1

[So, I imagine it like this, that the disclosure of information is very good and differentiated and intelligible, as in that a data protection person and an IT person explain where the risks and opportunities lie, and that a medically informed person tells me what the purpose of the whole thing is and also explains the process to me.... But for me, it is always important that the critical aspects are also highlighted. If there are advocates delivering propaganda [laughs] than I do not really feel well informed.]

Patient representative 1, translation by SW

Researchers also pointed out that compared to physicians, specifically trained personnel or reception desk personnel are better options for providing information in the informed consent procedure. The main reason invoked is the amount of time needed to communicate in person: scarce time for researcher physicians that ought to be used for what they deem to be more important tasks. Researchers assume that the amount of time needed to provide information in person will be huge, not only with regard to specific consent but also regarding broad consent. In addition to calling on other professions, researchers also mentioned web-based information and consent management platforms as possible sources of information that may help to reduce the amount of time that they must spend on data information and consent.

### Undue Pressure to Consent

Patient representatives referred to undue time pressure when data consent is tied to a clinical appointment. One representative referred to the mental overload of being asked to consent to health data research in a situation when patients seek treatment. Patient representatives generally agreed on the advantages of a data information and consent procedure scheduled independently of clinical appointments: at the clinic after treatment, at home before an appointment, or at home at a point of time completely at one’s disposal:

Und das ist letztlich auch eine Überforderung in dieser Situation, dass man wirklich noch gut abwägen könnte. Das heißt, man muss erst in einem Zustand sein der Ruhe oder der guten Überlegung, wo man wieder sein Hirn normal zur Verfügung hat, um dann zu entscheiden.

Patient representative 1

[And that is in the end also an overburdening, in that situation, that you could really weigh your options. I mean, one needs to be in a state of calm, or careful consideration, where you can access your brain normally, to decide.]

Patient representative 1, translation by SW

### Withdrawal of Consent

A topic that was discussed broadly in our interviews that has so far seen little attention in the ethical literature on consent concerns the implementation of withdrawal of consent. Researchers and data protection officers not only highlighted how important they consider this option to be but also pointed out that hospitals have not yet established smooth procedures to deal with withdrawal requests. Up until now, it has been difficult and time-consuming for them to handle such withdrawals. IT specialists were very much aware of this topic as well and highlighted that all digital solutions that are currently in development also aim to make changes to consent and withdrawal of consent transparent and easy to handle. From the perspective of patients, this topic was touched upon when they discussed whether and how their legal rights can be assured. Patient representatives were concerned that patients have no means to verify that their data have been erased (or samples have actually been destroyed) in response to their request:

Es muss ein Management geben, das die Einwilligungserklärungen widerrufbar macht. Wo geht der Widerruf ein von dem Patienten? Was wird mit dem Widerruf gemacht? Wie wird der umgesetzt? Wer ist dafür zuständig? In welcher Frist wird das gemacht? Was ist, wenn der Patient Auskunft haben möchte? Genau dasselbe Spiel. Eine Einwilligungslösung, eine Einwilligung bedeutet nicht ein Stück Papier, was im Schrank steht, abgeheftet wird und niemand hat damit mehr was zu tun, sondern all das müsste dann auch sichergestellt sein.

Data protection officer 2

[There needs to be a management, that allows for withdrawal of consent. Where is the withdrawal of a patient registered? What happens with the withdrawal? How is it implemented? Who is responsible for that? In what timeframe? What if the patient is asking for disclosure? The same thing again. A solution for consent, a consent, isn’t just a piece of paper that is put into the cabinet, filed away, and no one will ever interact with it anymore, but all of this needs to be ensured.]

Data protection officer 2, translation by SW

### Consent Management

IT specialists and the data protection officers agreed that not only for a model of tiered consent but also for the wide introduction of broad consent in German university medical centers, a digital system for consent management is required. Having to deal with a vast number of paper-based consent forms does not appear to be viable in any way. They pointed out that even in the case of broad consent, a given consent does not equal another given consent since, first, the MII broad consent offers options for patients to specify consent, and, second, the standardized MII consent sample text is regularly updated and thus comes in a number of versions that need to be tracked (as referred in the Attitudes on Specific and Broad Consent section). If any given consent is to be respected in regard to what it does or does not cover, a system that allows researchers or data trustees to access and manage consent forms is a necessity. Many of our interview partners opined that without this management, broad consent, or any other model of consent, cannot be handled efficiently. It was pointed out that the development of a software application for consent management would likely prove to be too expensive for smaller research projects since there are no such systems readily available on the market yet:

Nur ich sage mal, also das Medium an sich ist jetzt wahrscheinlich dann gar nicht so relevant, ob das auf Papier oder digital ist. Wichtig ist, glaube ich, nur, dass man größere Mengen dann einfach nicht mehr auf Papier händeln kann, was dann aber eher eine Rolle spielt in Richtung Datenherausgabe/Probenherausgabe und solche Sachen, dass man da einfach schon gezwungen ist, digital diese Consente abzubilden in irgendeiner Form.

Health IT specialist 2

[Well, I’d say, like, the medium itself probably isn’t all that relevant, whether it is paper or digital. What is important, I think, is that with larger numbers you cannot handle it on paper anymore, which in that case is more important in regard to handing out data or specimens and such things, that you are forced in that case, to map the consent digitally in some form.]

Health IT specialist 2, translation by SW

### Transfer of Health Data

Statements regarding the transfer of health data to for-profit organizations or non-EU countries revealed a dilemma. Concerns were voiced not only predominantly by patient representatives but also by the other groups that actors in the private sector have to be considered as less trustworthy. Industry partners cannot always be expected to act in the best interest of the public; data security standards might be lower in countries beyond the EU, and, therefore, patients should have the possibility to exclude the transfer of their data to industry partners or third countries:

Und ganz schwierig finde ich es, sage ich mal, wenn solche Daten halt, wie gesagt, an die Industrie weitergeleitet werden. Dann sind sie vollkommen außerhalb des Einflussbereiches der Uniklinika und so weiter und so fort. Und da kann man mir noch so viel andere Sachen halt erzählen, es geht in der Privatindustrie immer darum, Geld zu verdienen, immer möglichst viel Geld zu verdienen. Altruistische Motive sehe ich da in der Regel nicht am Werke.

Data protection officer 3

[And I think it is really problematic, let’s say, if such data, as we said, is handed over to industry. Then they are completely outside the area of influence of university medical centers and so on and so forth. And you can tell me different as much as you like, in the private industry, it is always about money. Making as much money as possible. I generally do not see any altruistic motivations at work there.]

Data protection officer 3, translation by SW

Regarding the contemporary research landscape, the necessity of exactly these kinds of cooperations was highlighted. The lack of permission to share data with non-European countries (or countries without an EU adequacy decision) was named as a showstopper for biobanks. As an example, the problematic legal status of the sensible wish of US-based companies to extract data from their robotic surgery systems used in German clinics in order to further develop their technologies was mentioned. It was pointed out that even publishing research results in US-based journals might be legally challenging, given the current regulation, if this requires uploading nonanonymized health data to US servers. With regard to broad consent in particular, a consent type that excludes these types of cooperation is regarded to significantly hinder valuable research:

Es gab, ich glaube, die Version 1.6D der Einwilligung...die aber die Einschränkung oder die wesentliche Einschränkung hatte, dass der Drittlandstransfer ausgeschlossen war, und dass eine Kooperation oder Zusammenarbeit mit Industrie ausgeschlossen war. ..., da kam für uns auch das Feedback: Ja Entschuldigung, damit, mit diesem Informed Consent können wir eigentlich im Grunde nichts anfangen oder sind extrem beschnitten.

Data protection officer 1

[There was, I believe, that version 1.6D of the consent...but that had the essential limitation that transfer of data to third countries was excluded, and that also a cooperation with industry was excluded....so we got the feedback: Well, excuse me, but ultimately, we can’t make use of this consent or we are extremely limited.]

Data protection officer 1, translation by SW

### Research Without Consent

One topic that was discussed controversially in regard to the legal framework regulating consent for health data research concerns the need for consent in general. Researchers voiced the opinion that collecting consent, no matter in which form, is a time-consuming process. They claimed that more research could be accomplished if research was possible without consent. While some researchers considered this as a desirable scenario, others highlighted the need to obtain consent and involve patients to ensure their continuous support. Beyond personal attitudes, all the researchers were pessimistic about research without consent becoming a viable option in Germany in the near future, notwithstanding the fact that the GDPR does allow for research without consent under certain conditions (compare with the information in the Background section):

Fände ich super. Das funktioniert nicht mit der Organspende und das funktioniert nicht mit dem Broad Consent für Forschung, weil da gibt es viel zu viele, die da... Nein, never ever. Aber finde ich gut.

Medical researcher 1

[That would be great. But that doesn’t work for organ donation and that doesn’t work for broad consent to research. Because, there are way too many.... No, never ever. But I think it would be good.]

Medical researcher 1, translation by SW

Referring to the GDPR, data protection officers did mention research options without consent. On the basis of specific state laws, local regulations exist which enable research without consent, most typically in cases of retrospective studies that only make use of health data stored at the hospital at which the research takes place (so-called *Eigenforschung*). However, the differences between federal state regulations were mentioned as a hindrance to multicenter cross-state research. A national regulation for health data sharing was suggested as a helpful means to achieve legal and governance clarity on this issue:

wenn Sie das mit einer Einwilligung versuchen, wird es immer Personen geben, die aus den verschiedensten Gründen sagen: Möchte ich nicht. Und wenn wir dann aber über ein gesundheitspolitisches oder gesellschaftspolitisches Gesundheitsmanagement sprechen, dann wäre es natürlich schon hilfreich, wenn unter klar definierten Rahmenbedingungen Patientendaten, also die digital vorhandenen Daten, für definierte Forschungsvorhaben verwendet werden dürften.

Data protection officer 1

[If you try that with consent, there will always be someone saying no, for different reasons. I don’t want to. And if we are talking about health policy, or sociopolitical management of public health, then it would of course be helpful if under clearly defined conditions patient data, data available in digital form, could be used for specific research purposes.]

Data protection officer 1, translation by SW

IT specialists were less concerned with this topic, but some voiced the opinion that any regulation that does not leave at least the possibility to opt out would stand in stark opposition to informational self-determination. This assessment was shared by some of the data protection officers.

This topic did not receive much explicit attention from patient representatives. However, when it was mentioned, it was not seen favorable at all. Options of control for patients in regard to the use of their data were rated as highly important, as expressed through a preference for models of specific or tiered consent, while even broad consent was rejected as not giving sufficient degrees of control:

wir reden ja zum Glück nicht um einwilligungsfreie Forschung, das ist ja nicht das Thema

Patient representative 2

[Luckily we are not talking about research without consent. It is not our topic here.]

Patient representative 2, translation by SW

oder dass man das [die Einwilligung] dann teilweise gar nicht braucht, gerade wenn man noch nach Europa schaut. Da werden ganz andere Sachen vorgeschlagen, inklusive Genom-Daten, auch der selbstverständlichen Freigabe ohne Rückholmöglichkeit. Also wenn wir jetzt schon in einem Ethikdiskurs sind, finde ich das irgendwie empörend.

Patient representative 2

[So that partially, it [consent] is not needed, in particular if we look at Europe. There are completely different things being suggested, including genomic data, as well as the releasing of data without an option to retract, as a matter of course. If we are already in an ethical discourse here, I find that somewhat outrageous.]

Patient representative 2, translation by SW

## Discussion

### Importance of Findings

In light of the current EU efforts to establish a European health data space and initiatives in other countries, including Germany, to develop digital infrastructures that allow large-scale access to health data for research, determining ethically, legally, and socially acceptable consent procedures is a task of utmost importance [1,3-8].

The attitudes expressed by our sample of German stakeholders provide some insight into how workable and acceptable consent may look like in Germany. As developments with regard to consent within the European health data space and in other countries are still in flux, these findings can prove valuable for other national contexts and the EU as well.

### Limitations

Our study has an explorative character, and the significance of the results is necessarily limited due to the small size of the study.

The stakeholders interviewed were limited to the 4 groups of medical researchers at university medical centers, health IT specialists, data protection officers, and patient representatives, all from Germany. With the European health data space in the making, interviews with stakeholders from other European countries on consent and models of consent would be of immense value. Perspectives from other stakeholder groups, such as for-profit health research companies, clinic administrative employees, and members of hospital executive boards, could provide further valuable insights.

Regarding patient attitudes, qualitative and quantitative studies on a larger scale would be needed to provide a clearer picture of interests and concerns.

### Options of Choice Within Consent

Our interviews do not reveal unequivocal endorsement of 1 specific model of consent across stakeholder groups. While researchers favored broad consent, patient representatives stressed that only specific consent opens up the possibility of receiving detailed information on planned research and the use of data within this research, with tiered consent being regarded as a possibly acceptable alternative. IT specialists and data protection officers were more neutral regarding this controversy. However, IT specialists pointed out that broad consent (at least as devised by the German MII) has limited advantages over tiered consent in regard to management effort as it comes in versions, contains optional modules for research locations, and provides some options for patients to tailor their consent.

In practice, with regard to implementation and management efforts, broad consent thus might often come close to tiered consent. If this holds true and if one wants to harness the advantage of tiered consent to be able to mirror patient preferences, more efforts should be made to determine which options of choice should actually be included. As it appears now, options are dictated mainly by legal requirements.

This issue appears important as patient representatives in our sample also raised concerns about for-profit organizations accessing their data. Currently, although the MII consent includes some options of choice for patients such as data being transferred to non-EU countries, data transfer to for-profit organizations is not among them. It is obligatorily included in the scope of the MII consent.

### Digital Tools and Platforms

There is no doubt that efficient data sharing across borders and between different institutions within the EU presupposes a well-functioning digital infrastructure for health data as well as consent data.

In addition, a variety of issues point toward including digital formats and tools as part of the information and consenting process as well. Researchers are reluctant to spend much time on informing patients and obtaining consent. Conflating clinical appointments with providing consent to health data research might result in mental overload and undue pressure. Patient information is often incomprehensible and does not enable patients to become well-informed. Digital tools could diminish researcher workload and, at the same time, provide patients with better opportunities to absorb relevant information. All these issues highlight the importance of adequate place, time, and format for providing information. Whether or not researchers should still be involved in this process in person remains a disputed issue.

Ensuring that withdrawal of consent can be handled smoothly and effectively is not as trivial as it may sound. Appropriate workflows and processes should still be implemented in many German hospitals. Digital consent management solutions will have to be able to deal with this challenge. Patient representatives in our sample doubted that it would be possible for patients to obtain information on which consent they have granted to an institution and which personal information is stored and used. They also expressed doubt whether a request for data erasure, a key GDPR right of persons whose data are used, would be fulfilled. These concerns and challenges could be mitigated by digital interfaces and platforms that are especially geared toward handling consent and patient requests concerning erasure, rectification, and withdrawal and are directly linked to the systems storing health data and processing research requests, as already in place in, for example, Estonia [43].

### Research Without Consent

The GDPR leaves room for the use of health data for research without consent (compare with the Background section). In specific cases and under specific conditions, German hospitals make use of this option. Due to the local character of these agreements and the diverse landscape of the federal state regulations in Germany, it is not transparent where and under which conditions such research takes place. In addition, the lack of unified regulation renders multicenter collaborations with centers in different states difficult. As a consequence, national regulations on health data research without consent appear desirable.

The stakeholder groups were divided on whether research on health data without consent could become more of a standard approach, compared with the few cases today. While data protection officers pointed out that the GDPR leaves room for this kind of research if national regulation is in place, medical researchers were pessimistic with regard to societal acceptance. Patient representatives in our sample were highly critical of this option.

Opt-out solutions, potentially tiered, in combination with easily accessible information about health data research and the possibility of checking for patients which studies make use of their data may be met with more acceptance since such a framework would be transparent and allow for a certain degree of control.

### Implementation of Consent and Consent Management

Implementation of the current procedures to obtain consent is criticized for various reasons, most importantly for lack of comprehensibility of information and implicit pressure to consent due to the integration of the data information and consent process in regular medical appointments. In addition, the impartiality of researchers and their competence regarding data protection are called into question. For researchers, providing information on data research is time-consuming and goes at the expense of other duties.

Detaching consent procedures from the clinical setting; providing additional schooled personnel for informing and obtaining consent; and providing alternative forms of information material, such as videos and well-structured web pages, seem promising avenues to mitigate the concerns and drawbacks of current consent implementation.

### Conclusions

Politically, in the EU and countries such as Germany, broad consent and, of lately, opt-out solutions are discussed and pushed forward as part of initiatives to build large health data research infrastructures. Our results indicate that these solutions as such may not do justice to the concerns and demands of patients. From the perspectives of participants in our study, both broad consent and opt-out solutions should include tiered options of consenting and opting out, respectively. These tiered options should mirror major patient concerns.

Regarding the implementation of consent procedures, including broad consent, digital management appears to be indispensable in enabling the handling of requests for information disclosure, erasure, rectification, and withdrawal of information. Participants in our study assumed that digital implementation holds great potential for improving workflows and diminishing researcher workload.

Participants in our study pointed out that having access to regularly updated information on ongoing studies is important. This issue should not be underestimated. It is relevant for both broad consent and opt-out solutions. Again, digital data management may allow the implementation of patient information modules that provide easily accessible, timely, and adequate information about ongoing research.

Finally, participants in our study stressed that it would be desirable to remove health data consent procedures from admission to the hospital and the clinical context in order to allow for a well-considered decision. Although digital consent management could allow for such a separation, implementation of consent in the German MII, for example, still connects hospital admission and consent.

## Appendix

Multimedia Appendix 1 Interview guide (German).

Multimedia Appendix 2 Overview of interview results (English version).

Multimedia Appendix 3 Overview of interview results (German version).

## Abbreviations

EU: European Union
GDPR: General Data Protection Regulation
MII: medical informatics initiative
