# Supplementary material for: Ethical, Legal, and Practical Concerns Surrounding the Implemention of New Forms of Consent for Health Data Research: Qualitative Interview Study
Source: J Med Internet Res. 2024 Aug 7;26:e52180. doi: 10.2196/52180 (PMC11339564; doi:10.2196/52180)
Supplement: Multimedia Appendix 1 [file jmir_v26i1e52180_app1.pdf]

Leitfaden zur Interviewstudie: Interviewstudie zu ethischen und rechtlichen Problemen der Umsetzung informierter Einwilligung zur Nutzung klinischer Daten für medizinische Forschungsprojekte

Einführung: Vorstellung von Modellen der Einwilligung

	studienspezifisch	gestuft („tiered“)	breit („broad“)
Papierform	SE	GE	BE
digital	DSE	DGE („dynamic tiered“)	DBE

Erläuterung:

Bei der studienspezifischen Einwilligung werden potentielle Studienteilnehmer:innen gezielt über ein bereits geplantes Projekt aufgeklärt und sie erteilen die Einwilligung zur Nutzung ihrer Daten allein in Bezug auf dieses Projekt.

Bei der breiten Einwilligung („broad consent“) werden feste und breit gefasste Ziele und Rahmenbedingungen möglicher, vielfacher, bisher nicht spezifisch geplanter Forschungsprojekte genannt, sodass die Einwilligung für alle Forschungsprojekte innerhalb eines benannten zukünftigen Zeitraumes gilt, die unter die genannten Ziele und Bedingungen fallen. Eine Zielformulierung könnte etwas die Nutzung auf medizinische Forschung eingrenzen, die der Erkennung, Behandlung oder Vorbeugung von Krankheiten dient. Die Medizininformatik-Initiative schlägt für die Gültigkeit einen Zeitraum von 30 Jahren vor.

Bei der gestuften Einwilligung („tiered consent“) werden Optionen benannt, die sich auf Ziele der Forschung, verwendete Methoden, beteiligte Einrichtungen und weiteres beziehen können, sodass Einwilligende durch das Ankreuzen verschiedener Optionen flexibel über die Enge oder Breite ihrer Einwilligung bestimmen. Die Daten könnten etwa zur Nutzung nur in EU-Ländern oder auch zur Nutzung für Partner außerhalb der EU freigegeben werden. Die Einwilligung kann auf eine spezifische Studie beschränkt bleiben, direkte Folgestudien mit einschließen, auf einen Forschungskontext wie etwa die Krebsforschung ausgedehnt werden oder auch generell Erforschung von Krankheiten umfassen. Eine Zustimmung zu den am weitesten gefassten Optionen entspräche in etwa einer breiten Einwilligung.

Etabliert ist die Einwilligung in Papierform, die üblicherweise im klinischen Kontext, nach einem persönlichen Aufklärungsgespräch erfolgt. Angedacht und in anderen Ländern getestet werden aber auch Modelle, bei denen die Einwilligung über eine digitale Plattform erfolgt („dynamic consent“). Auch hier ist jeweils ein Modell der studienspezifischen, gestuften oder breiten Einwilligung möglich. Der wesentliche Unterschied liegt nicht in den Optionen der Reichweite der erteilten Einwilligung, sondern im Kontext und der Flexibilität ihrer Einholung: Die digitale Einwilligung kann über ein Endgerät jederzeit und ortsunabhängig erfolgen und potentiell auch immer wieder neu angepasst werden („dynamic tiered consent“). In der Regel gehen diese Modelle mit der Annahme einher, dass auch die Informationsbereitstellung auf digitale Formate angepasst wird.

Fragen für alle Gruppen:

- Haben Sie Nachfragen zu den Erläuterungen der Einwilligungsmodelle?
- Die Modelle sollen unterschiedlichen Anforderungen gerecht werden. Sie sollen zum Beispiel Datenschutzvorgaben genügen, informationelle Selbstbestimmung ermöglichen, sie sollen Forschung an großen Mengen klinischer Daten nicht unnötig behindern, sie sollen praktikabel sein. Gibt es aus Ihrer Sicht weitere Anforderungen, denen die Modelle gerecht werden sollen?
- Welche Vorzüge sehen Sie hinsichtlich der genannten (und ggf. weiterer) Anforderungen in Bezug auf die vorgestellten Modelle?
- Welche Nachteile sehen Sie hinsichtlich der genannten (und ggf. weiterer) Anforderungen in Bezug auf die vorgestellten Modelle?
- Welche Risiken für Betroffene sehen Sie bei der Verarbeitung großer Mengen klinischer Daten?
- Welches der vorgestellten Modelle halten Sie alles in allem für am geeignetsten, um zukünftig die Einwilligung zu daten- und probenbasierter medizinischer Forschung angemessen umzusetzen?
- Wenn Sie keines der Modelle für geeignet halten, welche anderen Modelle oder welche andere Vorgehensweise jenseits der Einwilligung halten Sie für empfehlenswert?
- Abschlussfrage: Gibt es noch einen Punkt, den Sie dringend zur Diskussion ergänzen möchten?

Gruppenspezifische Fragen:

An Kliniken und in der Forschung tätige Informatiker:innen und Verantwortliche für IT

- Welches der vorgestellten Einwilligungsmodelle bringt Ihrer Ansicht nach in Bezug auf die Umsetzung an Kliniken besondere Vorteile oder Herausforderungen mit sich?
- Welches Modell hat spezifische Vor- und Nachteile bezüglich Datensparsamkeit, Datensicherheit und ähnlicher Datenschutzvorgaben?
- Welches Modell bietet die größten Vorteile oder Hürden bezüglich der Einführung einer einheitlichen Einwilligungslösung, wie z.B. im Rahmen der Medizininformatik-Initiative angestrebt?
- Welches Modell bietet die größten Vorteile oder Hürden in Bezug auf die Weitergabe von Forschungsdaten an andere forschende Einrichtungen?

Medizinisch Forschende

- Welche/s der Modelle ermöglicht Ihrer Ansicht nach eine angemessene Aufklärung über relevante Ziele, Chancen und Risiken der Forschung?
- Welches der Modelle ist am besten geeignet, zukünftige Forschung zu fördern? Worin liegen die Vorteile dieses Modells in dieser Hinsicht?
- Welches der Modelle würde die Forschung an klinischen Daten unangemessen behindern? Worin liegen die Hürden dieses Modells in dieser Hinsicht?
- Sind die Modelle im Hinblick auf die Frage, welche Formen der Datennutzung anschließend erlaubt sind für sie transparent? Wo gibt es ggf. Probleme?
- Wird aus Ihrer Sicht aktuell von Ethikkommissionen oder Datenschutzbeauftragten in angemessener Weise mit der Maßgabe, die Einwilligung Betroffener einzuholen und Datenschutzrechte Betroffener sicherzustellen, umgegangen? Sollten die Vorgaben liberaler oder strenger sein?

- In welchem Rahmen und zu welchem Zeitpunkt sollten PatientInnen Ihrer Ansicht nach idealerweise um eine Einwilligung gebeten werden? (Zuhause, in der Klinik – Anmeldung oder Behandlung, beim Hausarzt; mit qualifizierter Ansprechperson oder ohne?)

Verantwortliche Datenschutzbeauftragte von Kliniken, Universitäten und Ländern

- Welches der vorgestellten Modelle wird Ihrer Ansicht nach den rechtlichen Anforderungen der DSGVO (Art. 6 und 7, Art. 89, Erwägungsgrund 33, Art. 5, Erwägungsgrund 50 etc.), des BDSG und der Landesdatenschutzgesetze gerecht, welches nicht?
- Stellen die datenschutzrechtlichen Vorgaben Ihrer Ansicht nach ein angemessenes Schutzniveau für die Daten Betroffener sicher? Wo sehen Sie diesbezüglich Nachbesserungsbedarf?
- Wird mit dem Forschungsprivileg der DSGVO Ihrer Einschätzung nach in der Praxis angemessen umgegangen? Wird es zu eng oder zu weit ausgelegt, z.B. im Hinblick auf die erneute Einholung der Einwilligung für Forschungszwecke oder in Bezug auf Informationspflicht und Einschränkung anderer Rechte Betroffener?
- Wäre eine größere Vereinheitlichung der Einwilligungsverfahren (bundesweit im Rahmen der Medizininformatik-Initiative) aus datenschutzrechtlicher Sicht wünschenswert?
- Erleichtert oder erschwert eines der Modelle die Überprüfung der Einhaltung von Datenschutzvorgaben?
- Welche Lösung bietet Ihrer Ansicht nach die größte Transparenz für Betroffene?

Vertreter:innen von Patient:innenvereinigungen

- Werden die individuellen Rechte und Interessen von Patient:innen in den drei Modellen Ihrer Ansicht nach hinreichend gewahrt? Welches Modell hat hier Vor- oder Nachteile und welche Rechte oder Interessen sind da relevant?
- Geben die Modelle den Patient:innen hinreichende Möglichkeiten, ihre Daten für die Forschung zur Verfügung zu stellen, die sie fördern wollen?
- Erlauben die Modelle jeweils eine angemessene Aufklärung über die Ziele der Forschung?
- Erlauben die Modelle jeweils eine angemessene Aufklärung über mögliche Risiken?
- Bietet eine digitale Gestaltung der Einwilligung hier wesentliche Vorteile oder Nachteile?
- Stellen die jeweiligen Modelle eine Überforderung von Patient:innen dar (weil z.B. bei studienspezifischer Einwilligung zu oft gefragt werden muss oder bei gestufter Einwilligung zu viel Papier zu lesen ist oder weil bei digitaler Einwilligung die digitalen Medien zu nutzen sind?)
- In welchem Rahmen und zu welchem Zeitpunkt sollten PatientInnen Ihrer Ansicht nach idealerweise um eine Einwilligung gebeten werden? (Zuhause, in der Klinik – Anmeldung oder Behandlung, beim Hausarzt; mit qualifizierter Ansprechperson oder ohne?)
